# A Review of Sulfate Removal from Water Using Polymeric Membranes

**DOI:** 10.3390/membranes15010017

**Published:** 2025-01-09

**Authors:** Jamal Al Mehrate, Sadek Shaban, Amr Henni

**Affiliations:** Industrial Systems Engineering, Produced Water Treatment Laboratory, Faculty of Engineering and Applied Science, University of Regina, Regina, SK S4S 0A2, Canada; jma009@uregina.ca (J.A.M.); sms620@uregina.ca (S.S.)

**Keywords:** sulfate removal, membrane technology, water, modification and fabrication process, polymeric materials

## Abstract

Access to clean and reliable water has become a critical concern due to the global water crisis. High sulfate levels in drinking water raise health concerns for humans and animals and can cause serious corrosion in industrial systems. Sulfated waters represent a major challenge on the Canadian prairies, leading to many cattle deaths. While reverse osmosis (RO) membranes effectively remove sulfates, they are costly due to high-pressure requirements. Nanofiltration (NF) membranes present a more affordable alternative, outperforming traditional methods like adsorption, desalination, and ion exchange. Developing low-pressure ultrafiltration (UF) and microfiltration (MF) membranes could also reduce costs. This review explores advancements in polymeric materials and membrane technology to enhance sulfate removal, focusing on methods used to reduce fouling and improve permeate flux. Techniques discussed include phase inversion (PI), thin-film composite (TFC), and thin-film nanocomposite (TFN) membranes. The review also highlights recent fabrication methods for pristine and nanomaterial-enhanced membranes, acknowledging both benefits and limitations. Continued innovations in polymer-based membranes are expected to drive further performance and cost-efficiency improvements. This review found that studies in the literature dealt mainly with sulfate concentrations below 2000 mg/L, indicating a need to address higher concentrations in future studies.

## 1. Introduction

The United Nations World Water Development Report has stated that by 2050, water shortages could impact as many as 6 billion people. Approximately 12% of the global population lacks clean drinking-water sources [1]. Although water covers over 70% of the earth’s surface, 97% of it is too salty for human consumption. Most of the remaining 3% is underground or forms glaciers, leaving less than 1% available for human use [2]. Given the depletion of freshwater resources, there is an urgent need for more efficient and cost-effective water treatment methods to meet the ever-increasing demand for water. Water treatment systems such as ion exchange, distillation, and adsorption eliminate contaminants and produce pure water. Sulfate ions are common contaminants found in surface water, acid rain, acid mine drainage, and wastewater from industries such as paper, tannery, food production, oil refineries, and petrochemicals, which often discharge these ions improperly into the environment [3,4,5]. Consequently, remediating these effluents or contaminants is costly, potentially reaching millions of dollars, and poses significant management challenges. While sulfate is generally considered safe, high concentrations in water can negatively affect the environment, industry, and the health of humans and animals [6,7].

Over the past few decades, membrane-based technologies have gained favor due to their ability to efficiently separate substances at relatively lower costs and with increased ease of use [8]. Membrane materials are readily available and well-suited for large-scale applications [9]. There are various criteria for classifying membranes, such as the materials used in their fabrication (e.g., ceramic or polymeric); polymeric membranes include polyethersulfone (PES), polyvinylidene fluoride (PVDF), cellulose derivatives, polyacrylonitrile (PAN), polyvinyl chloride (PVC), polysulfone (PSf), poly(vinyl alcohol), and polyamide (PA). The sizes of their pores range from those associated with reverse osmosis (RO), nanofiltration (NF), and ultrafiltration (UF) to microfiltration (MF), and there are other distinguishing features [9,10,11]. Some challenges include the susceptibility to fouling, resulting in reduced flux and filtration capacity. Ongoing research actively explores ways to enhance polymeric membranes for sulfate removal and prevent fouling. For instance, recent studies focused on incorporating nanomaterials into polymeric membranes to boost hydrophilicity, permeability, selectivity, and thermal stability [12,13]. Techniques like adding nanomaterials are classified into four areas: (i) inorganic fillers, including different metal and metal oxide nanomaterials such as Al, Au, Fe, Ag, Mg, Al_2_O_4_, TiO_2_, Fe_2_O_3_, and SiO_2_; (ii) organic-based nanomaterials, excluding carbon materials such as dendrimers, cyclodextrin, liposome and chitosan micelle, N-halamine compounds, and polymer biomolecules; (iii) carbon-based nanomaterials such as single-walled carbon nanotubes, multi-walled carbon nanotubes, activated carbon, graphene, and carbon fiber; and (iv) composite-based nanomaterials that include combinations of metal-based, metal-oxide-based, carbon-based, and organic-based nanomaterials. They often have complex structures, such as metal–organic frameworks MOFs [14,15,16,17].

Different types of sulfates are found in the environment, such as gypsum (calcium sulfate), barite (barium sulfate), epsomite (magnesium sulfate), pyrite (iron sulfide), galena (lead sulfide), and mirabilite (sodium sulfate). The ability of sulfate compounds to dissolve in water changes depending on the presence of other chemicals. Magnesium, sodium, and potassium sulfate dissolve quickly in water. However, calcium sulfate (gypsum) has moderate solubility, and barium sulfate is usually insoluble, making it suitable for sulfate removal through chemical precipitation [18]. Raising water hardness by increasing Ca^2+^ and Mg^2+^ levels results in higher sulfate concentrations in the treatments because Ca^2+^ and Mg^2+^ come from CaSO_4_ and MgSO_4_ sources, respectively [19]. Higher levels of sulfate can increase the release of phosphorus into surface water. For instance, reducing sodium sulfate to sodium sulfide increases alkalinity, producing higher levels of phosphorus release in a lake [20].

**Figure 1:**
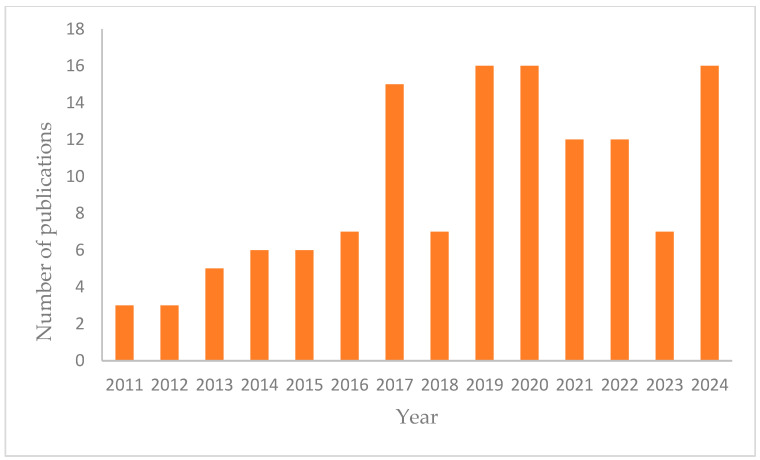
Number of publications per year, from 2011 to 2024, for treating sulfated waters using polymeric membranes; based on Web of Science research using these main keywords: sulfate removal, polymer membrane, and water.

Recent reviews discussed various methods for treating sulfated water. Researchers have yet to agree on the optimal technology for sulfate water treatment, as each method has advantages and disadvantages. Figure 1 shows that 132 studies have been published in the last two decades dealing with sulfate removal using a polymeric membrane. Publication numbers have increased steadily in recent years, which may indicate that it is a topic interesting to researchers, especially for questions involving high concentrations of sulfates in water.

The market for essential parts of RO water treatment systems is expected to grow from $11.7 billion in 2020 to $19.1 billion by 2025, with an annual growth rate of 10.3% [21]. The NF membranes market is set to grow significantly, increasing from $518 million in 2019 to $1.2 billion by 2024, with a robust annual growth rate of 18.2% [22]. The ultrafiltration membranes market is projected to increase from $4.4 billion in 2021 to $5.9 billion by 2026, at an annual growth rate of 5.9% from 2021 to 2026 [23]. The microfiltration membranes market was worth $3.9 billion in 2022 and is projected to grow to $6 billion by 2027, with a compound annual growth rate of 8.8% [24]. The treatment costs per cubic meter for removing sulfates were considered according to data analysed by Quintana-Baquedano et al. [25]; the study discovered that the cost for RO is $0.33/m^3^, and for NF combined with UF is $0.31/m^3^. These costs are significantly lower compared to the general costs of treatments, which are $1.17/m^3^, and $0.57/m^3^, respectively. The treatments indicated are generally more cost-effective than other sulfated water treatments like biological and precipitation treatments.

## 2. Polymeric Membrane Materials

### 2.1. Polyethersulfone (PES)

PES is known for its excellent mechanical and chemical stability, making it well-suited for demanding applications. It exhibits a high glass-transition temperature of over 225 °C, providing thermal stability even in wet and hot environments [26]. Despite their many advantages, one drawback of PES membranes is their inherently hydrophobic nature. Therefore, some studies have explored the formation of TFC membranes for ultrafiltration/nanofiltration, in which PES serves as the support layer [27]. In one study, PES membranes have undergone modification by incorporating an amphiphilic copolymer called Pluronic F127. This modification aims to improve the treatment of the water produced by enhancing the hydrophilicity of the membrane surface [28]. Research in blended membranes composed of PES/CA and polyethylene glycol (PEG) has shown that this material demonstrates excellent permeability, featuring a thinner outer skin layer, increased surface porosity, and larger pore sizes, with an average pore size of 0.15 µm [29]. A phase inversion method was used in the fabrication of PES/UF membranes in a hollow fiber configuration [30]. In a study by Li et al. [31], nanoparticles of TiO_2_ were used to improve strength and flux. In addition, PES can be fabricated by the electrospinning method [32,33]. PES membranes can also be manufactured by incorporating polyvinylpyrrolidone (PVP) as a pore-forming agent [34]. Moreover, PES membranes can be combined with different concentrations of chitosan to create PES/chitosan membranes. Their effectiveness in removing metals and sulfates from acid mine drainage (AMD) was evaluated. The authors concluded that chitosan holds promise for enhancing pure-water flux (PWF) and rejection. The maximum flux reached was 133 LMH, with a cation rejection level at 89% and 72% recorded for sulfate ions [35].

### 2.2. Polysulfone (PSf)

PSf membranes are available commercially and are primarily used for ultrafiltration. Studied types include cellulose and polyamide UF membranes [36]. A PSf membrane combined with polyvinylpyrrolidone and polyethylene glycol (PEG) as a modification gives a highly hydrophilic material and leads to fouling resistance [37]. Moreover, PSf is widely recognized for its exceptional resistance to highly acidic and alkaline environments and its thermal stability, allowing it to function effectively at temperatures up to 75 °C. However, it is essential to note that PSf polymers also exhibit a hydrophobic nature, which makes them highly prone to fouling [38]. Yadav et al. [39] studied PS and graphene oxide–vanillin (GO-vanillin) membranes designed to improve water purification. They found that a pristine PSf/vanillin membrane had a PWF of 39 LMH, and a PSf/(GO200-vanillin) had a PWF of 91 LMH. This enhancement was due to the increase in the concentration of graphene oxide (GO) in the membranes, enhancing their hydrophilicity and significantly lowering the water contact angle, from 61.4° in pristine PSf/vanillin membrane to 50.31° for the PSf/(GO200-vanillin) membrane. The optimized PSf/(GO150-vanillin) membrane exhibited rejection rates of 92.5% for 2000 ppm MgSO_4_. Tan et al. [40] created polyamide membranes with nanoscale Turing structures (a method of self-organization of molecules) based on interfacial polymerization (IP); the researchers incorporated PSf/PVA and applied piperazine (PIP) as activators and TMC as the inhibitor to form PA. They manipulated the reaction conditions to create membranes with diverse shapes, such as bubbles or tubes. These membranes demonstrate remarkable effectiveness in separating water from Na_2_SO_4_ and MgSO_4_, calculated at approximately 99.6% and 99.2%, respectively. Additionally, water permeability is notably high, with fluxes of 119 and 125 LMH. Another example is the use of a GO/PSf substrate and preparation of the PA selective layer, which was able to improve salt rejection of both Na_2_SO_4_ and MgSO_4_ and improve pure-water flux, at 95.2% and 91.1% and 2.4 LMH, respectively [41].

### 2.3. Polyacrylonitrile (PAN)

PAN is a polymer known to possess several advantageous characteristics, including robust mechanical stability, exceptional thermal and chemical resistance, and resistance to UV radiation. Additionally, PAN membranes have small pore diameters that can be controlled [42] and the membranes resist solvents [43,44]. Similar to PES, PAN is hydrophobic, which often leads to its being blended with hydrophilic polymers such as chitin or cellulose-based polymers to enhance its compatibility with aqueous systems [43,45]. However, PAN is naturally more hydrophilic than PES or PSf and possesses inherent antifouling properties [46], making it a popular choice for water filtration membranes [43,47,48]. A commercially available PAN/UF membrane which possesses a structure that is highly hydrophilic has been optimized for use at 45 °C [49]. Notably, by operating at a temperature higher than the typical range, the membrane exhibited improved resistance to fouling. An electrospun nanofibrous membrane was created by depositing polydopamine nanoclusters onto a crosslinked membrane composed of (PAN) and hyperbranched polyethyleneimine (HPEI) [50].

Nonetheless, one drawback of PAN is its limited solubility, as it can only dissolve in polar solvents like N, N-Dimethylformamide (DMF), N-Methylpyrrolidone (NMP), or *N*, *N*-Dimethyl-acetamide (DMAc). Nevertheless, PAN is well suited for fabrication techniques such as electrospinning or phase inversion. Yeh et al. [51] developed a TFC/PAN membrane by using electrospinning as a porous support and applying a thin layer of cellulose nanofiber followed by graphene oxide deposition. This layered structure aimed to improve filtration efficiency and enhance pollutant removal capabilities. Shahriari and Hosseini [52] designed and fabricated an NF membrane of PAN, citric acid, and TiO_2_ for water treatment. They found that increasing the concentration of TiO_2_ and citric acid led to enhancements in both permeate water flux and rejection, for the fabricated membranes compared to unfabricated membranes. In the best conditions, the experimental results showed a PWF at 130 LMH and a CaSO_4_ rejection rate of 82%.

### 2.4. Polyvinylidene Fluoride (PVDF)

Polyvinylidene fluoride (PVDF) is a hydrophobic polymer known for its excellent chemical and thermal stability and unique electrical properties [53]. These desirable characteristics have caused PVDF to be widely utilized in various membrane applications, including water and biomedical filtration [54,55], and it remains a valuable material in wastewater treatment [56]. However, PVDF showed low critical surface energy compared to PSf and PAN; this can lead to increased fouling and reduced water flux, limiting the lifespan of the membrane [57,58]. The enhancement of PVDF membranes by incorporating hydrophilic structures to promote water passage and reduce fouling has been extensively examined and discussed in a comprehensive review [57]. In other studies, surface hydrophilic modification was accomplished by employing tannin or polyethyleneimine [59]. As reported in various studies, membranes are commonly fabricated using the phase inversion method [60,61]. Incorporating the triblock polymers enhanced the performance of PVDF membranes, making them more suitable for applications where antifouling properties and efficient water flow are essential [62]. Mishra et al. [63] discussed the effectiveness of ferrous sulfide (FeS)- and carboxyl functionalized ferroferric oxide (CFFO)-incorporated PVDF-based nanocomposite membranes (PVDF/FeS/CFFO) for the removal of highly toxic heavy-metal ions from industrial groundwater. They concluded that the PVDF/FeS/CFFO membrane enhanced pure-water flux, at 1266 LMH compared to an unmodified PVDF value of 340 LMH. At the same time, the rejection of NaSO_4_ remained steady at around 98% for the first 40 min, but then declined to about 70% in the next 20 min. They found the conductivity decreased from 1678 μS/cm to a stable range of (3.9 to 6.2) μS/cm by collecting filtration every 5 min.

### 2.5. Polyvinyl Alcohol (PVA)

PVA is a polymer known to be soluble in water and for its hydrophilic nature, water permeability, antifouling potential, and thermal and chemical resistance. However, it is permeable to ions, prone to significant swelling, and compacts under pressure; when highly crosslinked, it exhibits a flux reduction [64]. As a standalone filter membrane, PVA must be crosslinked through covalent bonds [65]. This process introduces an additional parameter for optimization, namely, the crosslinking densities of PVA membranes. PVA is often incorporated into composite membranes with other polymers, like PAN, to enhance performance [48]. PVA contains hydroxyl and acetyl units, with the ratio determining whether it is classified as polyvinyl alcohol or polyvinyl acetate [66]. Hydrophilic semipermeable membranes are highly selective, enable high flux, and are optimal for water purification processes such as UF and RO [67]. PVA membranes can undergo crosslinking using organic chemicals such as aldehydes and organic acids such as glutaraldehyde or polyacrylic acid [68]. Alternatively, solubilization of PVA can be accomplished by acid-catalyzed dehydration with mineral acids like sulfuric acid or gelation using peroxidisulfates like potassium persulfate [69,70].

Additionally, incorporation of filler materials like zeolite can enhance PVA membrane performance by facilitating the passage of smaller molecules while restricting the flow of larger molecules [71]. Kim et al. [72] reported that sulfated zirconia incorporated in membrane preparation had a dual role as a filler material and an effective agent for crosslinking or insolubilization. The incorporation led to enhanced and adjustable membrane performance, specifically regarding permeation rate and selectivity. Zhang et al. [73] created a PVA/mercaptopropyltriethoxysilane 0.6 (MPTES)/TFC membrane. The study reveals that the rejection and water flux can be controlled by adjusting the MPTES content in the coating solution. The optimal membrane exhibits a Na_2_SO_4_ rejection rate of 97.2% and a water flux of 11.6 LMH, and this increases to 98.0% rejection and 43.3 LMH water flux after oxidation by H_2_O_2_, due to the Donnan effect, a name given for the behavior of charged particles near a semi-permeable membrane that sometimes fail to distribute evenly across the two sides of the membrane. Additionally, the membrane exhibits excellent pH stability and maintains a Na_2_SO_4_ rejection rate above 95% even after exposure to 15% H_2_SO_4_ or 4% NaOH solutions for 30 days, which can ensure the appropriate water flux.

### 2.6. Cellulose Acetate (CA)

CA membranes possess desirable characteristics such as solid hydrophilicity, enhanced water permeability, and reduced susceptibility to membrane fouling [74]. Natural polymers, including cellulose and chitin, are highly suitable for water filtration due to their inherent hydrophilicity and widespread availability [75,76]. They are typically used in regenerated or derived forms or processed using specialized casting and spray-coating methods [75,76,77,78]. These polymers are made from copolymers such as PES/CA and PAN/CA, or applied as a barrier layer on more easily processable supports [76,79]. On the other hand, they are sometimes chemically modified with materials such as cellulose acetate and chitosan, exhibiting enhanced solubility, and can undergo processing techniques like electrospinning or phase inversion [47,80,81]. Many products today contain cellulose or its variants, making it a common choice for commercially available membranes, especially for RO support materials [56]. Ounif et al. [82] carried out similar work and used a phase inversion to create a CA/NF membrane and evaluate its functionality. The study examined the determinations of water permeability, contact angle, and the rejections of salts such as Na_2_SO_4_ and CdSO_4_. They noticed that increasing CA concentration reduced the membrane porosity, meaning that decreased water permeability and a reduction in the contact angle from 76° to 47° were observed, but it enhanced salt retention. The results demonstrate that the rejection levels of Na_2_SO_4_ and CdSO_4_ were at 81.3% and 90%, respectively. This enhancement is due to the divalent ions having larger hydrated sizes, while the PWF increased linearly as the transmembrane pressure (TMP) increased, as described by the Hagen–Poiseuille equation.

### 2.7. Polyamide (PA)

PA membranes with high strength and durability properties are employed for purifying water. Most of the typical commercially used membranes, including RO, NF, and UF membranes, are TFC membranes composed of two or three layers produced through interfacial polymerization [83]. The process of obtaining fresh water from seawater and wastewater depends on the selective permeation of water through the polyamide layer [84]. Therefore, the permeance and selectivity of polyamide composite membranes depend on their structural morphology and the quality of the ultrafiltration support [83]. Globally, researchers have focused on modifying the surface of the PA/NF TFC membrane to reduce fouling caused by organic and biofouling in the feed water. For example, Baige et al. [85] proposed a technique that involves the deposition of multiple layers of polyelectrolytes onto the NF membrane surface. This modification strategy aims to improve the ability of the membrane to resist fouling. The results showed a 15% improvement in fouling resistance, with a rejection of 98% for MgSO_4_. Another work by Yuan et al. [83] presents a novel asymmetric PA nanofilm consisting of two layers: a spherical polyamide dendrimer porous layer and a polyamide dense layer with highly ordered nanovoids. The nanofilm is formed initially by covalently attaching the dendrimer porous layer onto a PSf support surface through a diazotization coupling reaction. The membrane demonstrates significantly improved water flux, at 270 and 264 kg m^−2^ h^−1^, higher than a traditional polyamide membrane at 68.5 and 71.4 kg m^−2^ h^−1^, and achieves divalent rejection rates for MgSO_4_ and Na_2_SO_4_ as high as 99.1% and 99.2%, respectively. In a study by Karabacak et al. [86], the efficiency of three commercially available nanofiltration PA/NF membranes (DK-NF, NF270, and DL-NF) was evaluated for their ability to remove sulfates from drinkable surface water at a content range of 370–460 mg/L. The results showed that the NF270 and DL-NF membranes achieved over 98% sulfate rejection, whereas the DK-NF membrane provided an 82% rejection rate. Both NF270 and DL-NF membranes also exhibited excellent flux recovery after chemical cleaning. Based on these findings, the NF270 membrane emerged as the best-performing membrane among the three tested.

## 3. Modification and Improvement in Polymeric Membranes

**Figure 2:**
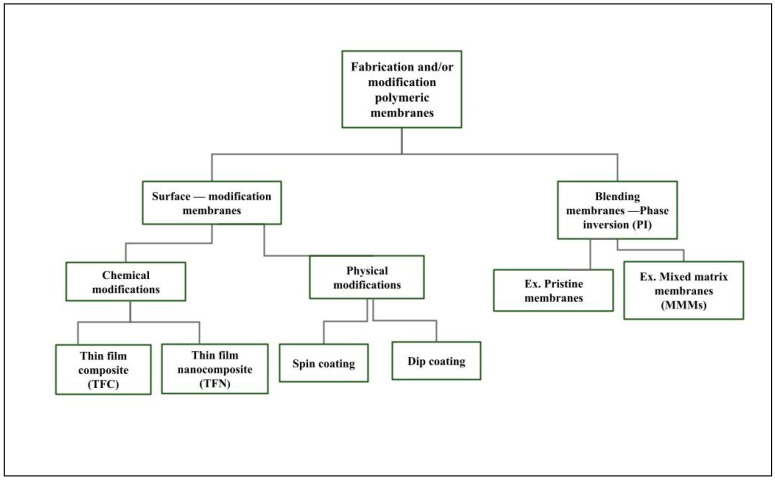
The general process for the fabrication and modification of polymeric membranes (adapted from [101]).

Polymeric membranes are well-known for their high selectivity and adaptability to specific process conditions. They are the favored technology among industries for their affordability, low energy consumption, and high effectiveness in dealing with sulfate water treatment. Membranes have been developed with applications that selectively allow specific ionic solutes to pass from the feed water into the filtration [87]. Solutes are rejected through a combination of exclusion and transport mechanisms, such as steric hindrance, Donnan exclusion, and dielectric exclusion [88]. In some cases, solute rejection occurs because the solutes adhere to the membrane surface [89]. The contact angle with water indicates how wettable a membrane surface is. It ranges from the super-hydrophilic contact angles close to 0° to the super-hydrophobic contact angles above 150°. Figure 2 shows the most basic processes for the fabrication and modification of polymeric membranes, including their improvement. The work published by Kim and Bruggen [90] discusses how nanoparticles like nano-TiO_2_, nano-alumina, silver, silica, zeolites, and carbon nanotubes improve polymer membranes. They concluded that membranes modified with nanoparticles experienced reduced fouling. Specifically, membranes with titania were most effective in lowering the fouling caused by organic solutes in polymeric membranes.

Furthermore, nanoparticles can modify membranes, depending on the specific functional groups needed. However, there is concern about nanoparticles’ potential ecotoxicity [90,91]. In a study, Ba-Abbad et al. [92] discussed the enhancement of polysulfone (PSf) membranes by the addition of hydrophilic cobalt-doped zinc oxide (Co-ZnO) nanoparticles. The study highlights the effectiveness of (Co-ZnO) nanoparticles in reducing the contact angle values from 82° to 62° and increasing the water flux. This membrane achieved a rejection rate for Na_2_SO_4_ of about 55%. Wu et al. [93] created a TFN membrane using PSf as a support layer, incorporating mesoporous silica nanoparticles into a polyamide matrix through interfacial polymerization. They found that a covalent bond forms between the silica nanoparticles and the active layer of the TFN membrane. This resulted in membranes with increased pure-water flux values, reaching 32.4 LMH, which is approximately 1.5 times higher than the traditional thin-film composite membranes, while maintaining high rejection rates for Na_2_SO_4_ of above 80%, but showing a lower rejection for MgSO_4_, at just above 30%. The thin-film nanocomposite membrane displays better resistance against fouling and demonstrates satisfactory long-term stability.

Agboola et al. [94] considered the performance of two nanofiltration membrane brands, Nano/Pro/3012 and NF90, in cleaning water of contaminants. They found that higher pH values led to increased removal of cations in both membranes, as lower pH could cause fouling. On the other hand, better removal occurs at a lower pH for anions. It is crucial to strike the proper pH balance for effective operation. The rougher membrane, NF90, outperformed Nano-Pro-3012 in removing sulfate. Using a water sample from the western Gauteng basin region of South Africa containing 3500 mg/L of sulfate, NF90 removed 97.6%, and Nano-Pro-3012 removed 86.3%, of the sulfate at a pH of 2.2. Juholin et al. [95] conducted an experiment applying a ZnO coating using atomic layer deposition to a commercial NF membrane NF270. They noticed a reduction in reversible fouling, though irreversible fouling remained unaffected, and there was a potential slight increase in the relative flux compared to membranes without the coating, specifically, NF90 and NF270. However, they observed that some Zn from the coating leaked into the treated water, while the membrane’s ability to reject sulfate remained consistent, at over 91%.

Al-Nahari et al. [96] studied a high-flux sulfonated polyamide thin film composite nanofiltration membrane, the thin film composite/benzidinedisulfonic acid (TFC/BDSA). The membrane was fabricated by combining aqueous monomers 2, 2-benzidinedisulfonic acid (BDSA) with piperazine (PIP), and organic monomer trimesoyl chloride (TMC) with triethylamine (TEA) as an aqueous catalyst. Results showed that BDSA and TEA had a synergistic effect on enhancing the performance of the membrane. The TFC/BDSA membrane achieved a Na_2_SO_4_ rejection rate of 99.6%, which was over 2.0% higher than the pristine membrane. Additionally, the flux recovery ratios for the (TFC/BDSA) membrane, after two cycles of filtration and cleaning, were 101.1% and 99.5% when fouled with sodium alginate and humic acid, respectively. Wang et al. [97] noted a rise in permeate flux and improvement in salt rejections, reporting the highest water flux as 38.91 LMH. In contrast, 87% of Na_2_SO_4_, 72% of MgSO_4_, and 24% of NaCl were rejected by incorporating acid-functionalized carbon nanotubes (CNTs) into polyethersulfone (PES). As the concentration of CNTs increases, the contact angle of the mixed matrix membranes (MMMs) decreases compared to pristine PES membranes. The change of contact angle was from 62.72° for a pristine PES membrane to 53.22° for PES/CNT. Qu et al. [98] reported on a membrane’s rejection based on surface functionalization through epoxy, amine, and sulfonic acid. The surface functionalization acts as neutral, positive, and negative charges. The results indicate that magnesium chloride and sodium sulfate have the lowest rejection, with up to 87% rejection of magnesium chloride and 90% rejection for Na_2_SO_4_, respectively. Alam et al. [99] synthesized a (PES/Fe_3_O_4_) mixed matrix nanocomposite membrane and used it for water purification. They treated 1000 mg/L MgSO_4_ and NaCl to study the membrane pure water flux and rejection. The membrane exhibits the highest pure water flux at 15% Fe_3_O_4_, while the highest rejection occurred at 10% Fe_3_O_4_ for MgSO_4_ and NaCl, about 82% and 68%, respectively. They also found that the contact angles reached 57.7° and 58.4°, respectively. Finally, Kong et al. [100] fabricated TFN membranes. The development focused on the membranes incorporating amine-functionalized Single-Walled Carbon Nanotubes (NH2-SWCNTs) through a process known as interfacial regulation. The goal is to enhance the performance of the TFN membranes for water purification applications. This study found that membranes containing 0.002 wt% of (NH_2_-SWCNTs) demonstrated exceptional water permeability, reaching a flux of up to 17.8 LMH, 71.1% higher than the pristine membrane. Additionally, these membranes achieved high rejection rates, with 91.0% for MgSO_4_ and 96.34% for Na_2_SO_4_.

## 4. Modules of the Membranes

**Figure 3:**
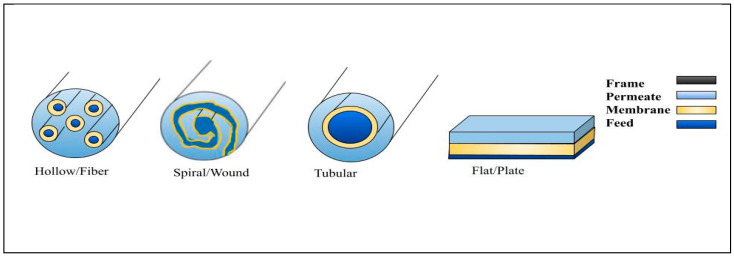
Four membrane module types (adapted from [108]).

Four primary types of membrane modules are typically available: spiral/wound, flat/plate, tubular, and hollow fiber; these are illustrated in Figure 3. Flat/plate and tubular modules are suitable as prototypes for studying sulfate removal (small-scale pilots) such as those used in laboratory-scale investigations, due to ease of preparation and versatility [102]. Hollow fiber and spiral/wound modules are commonly applied in large scale pilots such as those involving offshore refineries, brackish water, seawater, and wastewater operations; they are utilized for the reduction and removal of sulfate salt and various waste. The module selection is based on operational factors and economics [102]. Table 1 summarizes the properties of multiple types of membrane module designs. The most used module in industrial applications for NF or RO membranes is the spiral/wound module. This module comprises a flat sheet membrane wrapped around a perforated permeate collection tube. The feed flows on one side of the membrane, while the collected permeate spirals towards the center collection tube on the other side [103]. However, these modules are relatively costly because of their intricate design and engineering requirements. Spiral/wound modules offer a gentle flow over the membrane surface. Still, they are not widely used in MF and UF systems for wastewater treatment as they cannot be back-washed, and require extensive pretreatment [104]. Flat/plate modules consist of flat sheets of membrane organized into plates stacked within a framework. Patterned spacers prevent membrane adhesion and create channels for feed and permeate flow. They are utilized for highly suspended solids because of their ease of cleaning, but they can be expensive due to the large amount of hardware required and the small membrane area [104,105]. In tubular modules, the membrane is typically located inside a tube, and the feed solution is pumped through it. They are cost-effective and have a long lifespan. However, tubular membrane systems consume 50% more power than those based on hollow fiber or spiral/wound modules. They are often favored over hollow fiber membranes as they experience less severe fouling [103,106]. Hollow fiber modules utilized in seawater desalination comprise bundles of hollow fibers enclosed in a pressure vessel. These modules are designed with either a shell-side feed configuration, where the feed solution flows outside the fibres and exits through the fibre ends, or a bore-side feed configuration, where the feed circulates through the fibers. They are widely used in MF and UF membranes, and a key advantage is their ability to be cleaned using backwash by changing the direction of the permeating stream. Additionally, hollow-fiber modules have a high packing density, making them efficient in various filtration processes [103,107].

## 5. Mechanisms of Membranes

**Figure 4:**
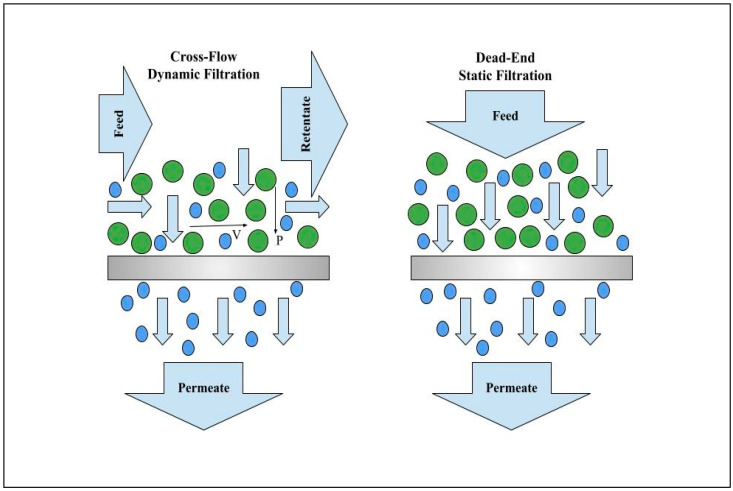
Description of how membranes work (Green bubbles: oil, Blue bubbles: water) (adapted from [110]).

Membranes work in two ways: cross-flow and dead-end. In cross-flow, the liquid moves next to the membrane, and in dead-end, it goes straight onto the membrane surface, as illustrated in Figure 4. The advantage of using cross-flow, as opposed to to dead-end, in sulfate water treatment applications is the enhancement of the lifespan of the membrane by reducing fouling through mechanisms such as shear diffusion, drag force, and internal lift. This helps reduce concentration polarization [109].

As shown in Table 2, different membranes exhibit specific performance characteristics that make them commercially viable.

**Figure 5:**
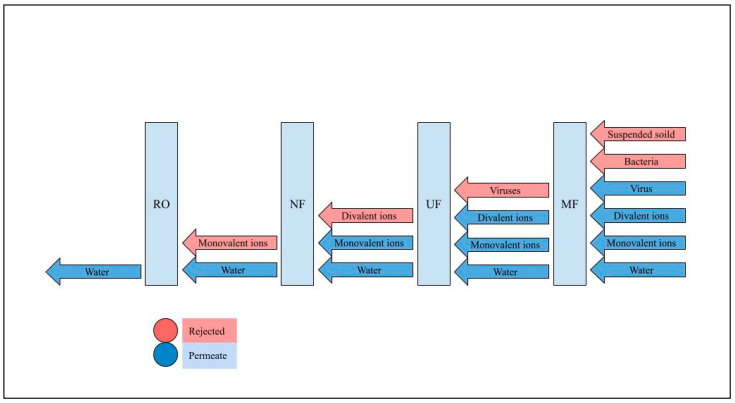
Filtration process in different membranes.

Each membrane is designed to target specific types of contamination, based on its respective capabilities and properties. Figure 5 illustrates the filtration process in different membranes. In response to freshwater scarcity, polymeric membrane technology, particularly NF and RO membranes, has emerged as a promising solution for sulfate removal. Table 3 shows commercial membranes which are recommended and tested by companies for sulfate removal.

RO removes over 98% of the monovalent and divalent ions. NF membranes generally exhibit high rejection of divalent ions, at over 95%, while monovalent ions can be removed across a wide range, with rejection rates ranging from around 20% to 80% [89]. Table 3 lists some commercial polymeric membranes utilized for sulfate removal from water and recommended by various companies; Table 4 and Table 5 list some commercial and fabricated membranes used for sulfate removal. The tests conducted by the companies used spiral wound modules for high-pressure results, while low-pressure tests were performed using flat modules. Multiple factors, such as diffusion coefficient, porosity, membrane pore diameter, and solute size, influence membrane operation [112]. The mechanisms involved in the retention operation include size exclusion, solute–membrane interactions, and differences in solute diffusion rates [113]. The molecular weight of a solute is often used as an indicator of size, but other parameters, like Stokes’s radius or equivalent molar diameter, can also be considered [114].

**Table 3:** Commercial membranes recommended and tested by companies for sulfate removal.

Companies	Type/Polymer Material	Solute	Rejection %	Pressure	Refs.
TriSep	UA60/Polypiperazine amide, SB90/Cellulose Acetate	MgSO_4_	70, 97	8 bar	[109,115]
GE	CK/Cellulose Acetate, HL/Polyamide,	Na_2_SO_4_ MgSO_4_	92	15 bar, 8 bar	[109]
Dow	NF, NF90, NF270/Polyamide	MgSO_4_	99	9 bar	[109,116]
Veolia	DK, RL/Polyamide	MgSO_4_	96, 98	7 bar	[117]
Synder	NFX, NFW/Polyamide	MgSO_4_	97, 99	8 bar, 7 bar	[109,117]
Microdyn Nadir	NP010, NP030/Polyethersulfone	Na_2_SO_4_	35–75	40 bar	[109,117]
Alfa	NF, NF99HF, RO90, RO99/Polyester	MgSO_4_	99, 99, 90, 98	5 bar, 9 bar	[118]

**Table 4:** List of research efforts using commercial membranes for sulfate removal *.

Membranes	Materials	Operation Conditions	Solution Type	Rejection%	Flux LMH	Refs.
MPS44 NF70 DESAL	Org.Selro PA PA	8 bar, 20 °C, 5–200 mg/L, pH 6	Na_2_SO_4_ and nitrates	85–66 94–91 60–45	8 71 50.5	[119]
Hydr70p TNF270	SPES PA	8.3–20 bar, 25 °C, flow 14.3 L/min, pH 2–2.8	Na_2_SO_4_	89 75	2.8–3.6 2.9–4.1	[120]
NF90 NF200 NF270	PA	6–22 bar, 25 °C, 340 L/h, 1780 mg/L,	Secondary effluent	75 60 65	8 22 35	[121]
NF90 NF270	PA	5–20 bar, 28 °C, pH 7	Na_2_SO_4_	96 88	-	[122]
NF90 NF270	PA	4–9 bar, 25 °C	Na_2_SO_4_	66.586.5	2.2 41.5	[123]
TFC-SR NF70 NF90	PA	5–20 bar, 25 °C	Na_2_SO_4_	96 99 93	12.3 2.6 3.6	[124]
NF Desal DK	PA	1–25 bar, 25 °C, flowrate 1800 L/h	MgSO_4_ Na_2_SO_4_	98 99	-	[125]
Toray T610, NF 270 NF Desal 5 L	PA	6–15 bar, 2000 mg/L	MgSO_4_	94 91 94	205 143 80	[126]

* Most experiments were performed at 25 °C.

**Table 5:** Polymeric membrane-based studies of sulfate removal from water.

Membrane Materials	Operation Condition	Solution Type	PWF or Flux (LMH)	Rejection%	Refs.
PSf + PVA + silica	10 bar, 23 °C	NaSO_4_	61.9	97.5	[127]
PES + PA + TiO	6 bar, 25 °C	MgSO_4_	9.1	95	[128]
PES + PA + Ag	14 bar, 25 °C	MgSO_4_	92	97	[129]
PES + chitosan + multiwalled carbon nanotubes (MWCNTs)	2–10 bar, flow rate 16 L/min pH 6.4,	NaSO_4_ MgSO_4_ NaCl	15.50	89.05 66.74 50.89	[130]
PSf + GO	4 bar, pH 2–12	Na_2_SO_4_	**-**	72%	[131]
PSf, + MWCNT + Ag	14 bar, 23 °C, pH 7	Na_2_SO_4_ NaCl	**-**	95.6 88.1	[132]
PAN+ chitosan	2–12 bar, 30 °C	Na_2_SO_4_ ZnSO_4_ CuSO_4_	18.35	97.2 ~92 ~89	[133]
PAN+ HACC	5–14 bar, 25 °C	Na_2_SO_4_ MgSO_4_ K_2_SO_4_	13.6	~28 ~35 ~20	[134]
PSf + PA + SPES	5 bar, 25 °C, flow feed rate 7 L/min	Na_2_SO_4_ MgSO4	128.8 115.2	99.4 96.5	[135]
PVDF + CMC + ZnO	10 bar pH 6	Na_2_SO_4_ MgSO_4_	139.7	95.01 90	[136]
PPEA + TFC	10 bar, 80 °C, 2000 mg/L	Na_2_SO_4_	400	96	[137]
PSf + HNT	9 bar, 2000 mg/L	MgSO_4_	30	94.4	[138]
PES + silica+ BHTTM	6 bar, 25 °C, 2000 mg/L, Na_2_SO_4_, MgSO_4_, pH 7	Na_2_SO_4_ MgSO_4_	15.21	85 ~57	[139]

Most experimental studies dealt with a solute concentration of 2000 mg/L NaSO_4_ or MgSO_4_ at 25 °C.

## 6. Fabrication Techniques

A handful of studies are highlighted in Table 5 in the previous section, focusing on fabricating, testing, and applying various polymeric materials for sulfate removal from water. The fabrication technique discussed in this review is divided into two processes: (i) blending membranes and (ii) surface modification membranes. The blending process creates membranes by phase inversion, denoted as phase separation (PS). A description of PS is presented in the next section. Nevertheless, electrospinning, additive manufacturing, and other fabrication approaches can enhance and adapt polymeric membranes. Despite the different techniques, the ultimate objectives remain consistent, namely, minimizing fouling and enhancing membrane functionality.

### 6.1. Phase Inversion (PI)

**Figure 6:**
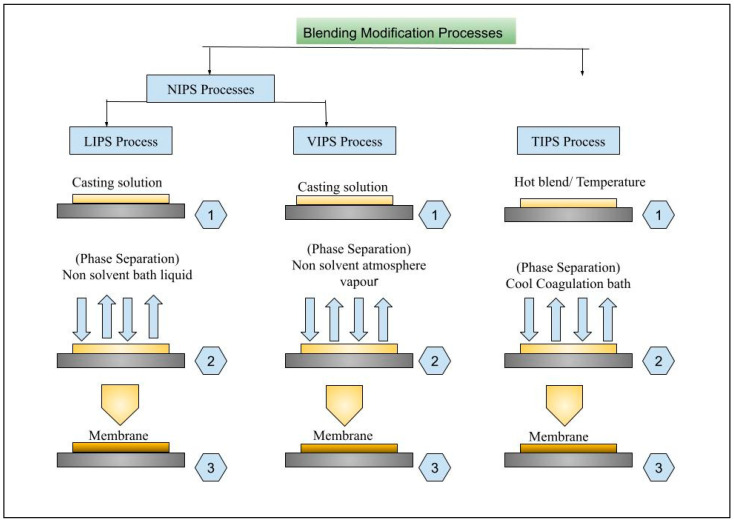
Different types of phase-inversion processes (adapted from [149]).

In a pioneering study, Loeb and Sourirajan introduced the concept of phase inversion in membrane technology during the 1960s [140]. Since then, it has become a significant development in the field. This method involves three components: polymer, solvent, and non-solvent [141]. It is known as non-solvent-induced phase inversion (NIPS). It is the primary method used in industry to produce asymmetric structures and polymeric membranes, in which a homogenous polymer solution is placed in a coagulation bath and converted into two phases. One of them is the polymer-rich phase, which forms the solid skeleton of the membrane, while the liquid-rich phase contributes to the creation of pores. The resulting structure typically consists of sponge-like or finger-like macro- or micro-pores [103,141]. Figure 6 presents the different types of phase inversion methods. NIPS can be divided into liquid-induced (LIPS) and vapor-induced (VIPS). In the LIPS method, a mixture of polymer, solvent, and volatile non-solvent is spread on a porous support using a doctor blade. In particular, ElGharbi et al. [56] describe the process known as tape or knife casting. The polymer solution is applied to a suitable support, such as a non-woven fabric, and then immersed in a non-solvent bath, usually DI water. The solvent enters the non-solvent, while the non-solvent penetrates the polymer solution. This results in the rapid formation of a solid membrane quite often utilized for UF applications [66,142]. VIPS, or precipitation from a vapor, was first introduced by Zsigmondy and Bachmann in 1918 and further developed by Elford in 1937 [143,144]. In VIPS, the membrane is cast and placed in a chamber with non-solvent vapors. These vapors enter the polymer solution and cause phase inversion, which is usually prepared using MF. VIPS is known for controllability but takes more process time [145,146]. Third, thermal precipitation or thermally induced PS (TIPS) is used for polymers that cannot dissolve at room temperature. The polymers are, therefore, dissolved in solvents at high temperatures, and a cast is applied as a support. As the temperature is reduced, precipitation occurs. Methods like evaporation, extraction, or freeze-drying remove the solvent in IPS. This method offers advantages such as a simple process, reliable results, minimal defects, and the ability to create highly porous materials [141,147,148].

### 6.2. Surface Modification Membranes

Two primary solutions were proposed and applied for the surface modification of membranes: chemical and/or physical modifications. The latter is carried out by coating the surface, preparing thin-film membranes, a process which will be described in the next section.

#### 6.2.1. Physical Surface Modification

##### Dip Coating

**Figure 7:**
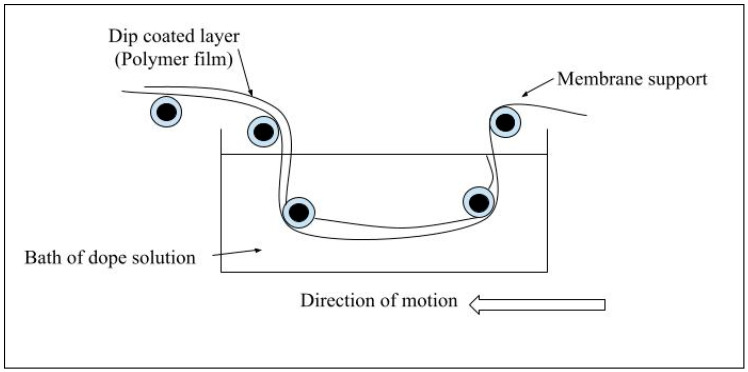
Description of the dip-coating process (adapted from [56]).

Dip coating is a process widely used for preparing thin-film polymeric membranes in various industries and laboratories due to its low cost and straightforward design. The process is illustrated in Figure 7 [56]. Initially, the substrate is immersed in a polymer solution for a specified duration. Afterward, the substrate is withdrawn, allowing the excess solution to drain off by gravity. This method may not be suitable for applications where coating is needed on only one substrate side, as the solution typically covers the entire submerged surface [150].

##### Spin Coating

**Figure 8:**
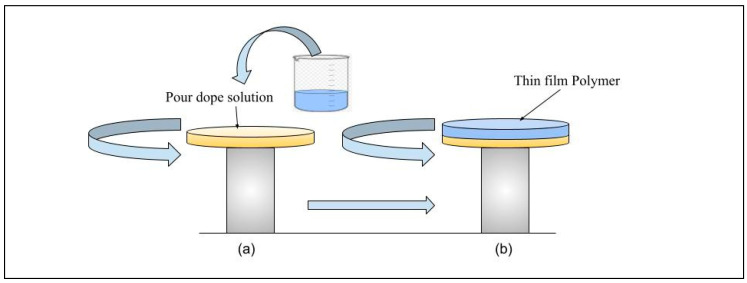
Description of the spin-coating process: (**a**) applying a polymer solution to a substrate; (**b**) spinning (adapted from [56]).

The spin coating process for membrane fabrication involves several key steps, as illustrated in Figure 8 [56]. Initially, the polymer solution is deposited onto the substrate surface. The substrate is spun using centrifugal forces to evenly spread the solution and remove excess. The speed and duration of spinning are crucial, as they determine the film’s thickness, with faster speeds typically producing thinner films. The solution thickness decreases as spinning continues, until a stable, uniform layer is achieved [150]. Finally, dip and spin coating membranes can undergo a phase inversion to achieve their final structure.

#### 6.2.2. Chemical Surface Modification

Chemical surface modification enhances membrane purification performance. These membranes fall into two categories: (i) thin-film composite (TFC) membranes, which are created through a unique process called interfacial polymerization (IP) in order to enhance their properties; and (ii) thin-film nanocomposite (TFN) membranes, which are developed and improved by adding particles to separate dyes and sulfate salts.

##### Thin-Film Composite (TFC)

TFC membranes differ in their chemical structures and how they are put together. Fabrication usually starts with a very porous base, which then has a thin solid layer of a different material added on the top. Polymers commonly form the support layers by phase inversion, the addition of coatings, the use of plasma, or the treatment of the surface using IP [151]. In the traditional synthesis method, a bifunctional amine is dissolved in water, and a trifunctional acid halide is dissolved in an organic solvent which is chosen for its immiscibility with water. The support membrane is first soaked in the aqueous solution and then transferred to the organic phase after saturation. Polymerization occurs between the monomers in the organic phase, forming a thin layer on the membrane [151]. TFC/RO membranes first came into the industry in 1972. These membranes are based on unique materials called aromatic polyamides, and worked better than the earlier CA membranes. In processes like NF or RO, about 50 to 90% of the water becomes pure (soft) water, and the rest becomes concentrated or rejected. This wastewater needs extra treatment and care [152,153]. The most commonly used materials for NF or RO membranes are cellulose acetate and polyamide composites [152] and modified forms of UF membranes like sulfonated polysulfone [154]. Moreover, the critical difference is that CA membranes resist fouling better, while polyamide composites usually have higher rejection rates [152]. Additionally, polyamide composite membranes have a negative charge, making them better at rejecting sulfate than are neutral-charged cellulose acetate membranes [152]. For instance, when using a solution with 2000 mg/L of salt at certain conditions, the TFC membrane shows a high flux of 46 LMH, compared to the CA membrane with 37.4 LMH. The rejection is about 99.5% for salts with TFC, compared to 97.5% for CA [154]. The efficiency of these membranes is influenced by their thermal, mechanical, and chemical properties. Chemical modifications can be made to change their surface charge, water-attracting ability, roughness, or permeability for better performance. A special IP method is used to make a TFC membrane. Cadotte [154] patented the TFC in 1981. The membrane was used to treat water containing many salts at 35,000 mg/L and at a high pressure of 103 bar. It exhibited a rejection of 99% salts and a flux rate of 31 LMH. Modern membranes do much better now. They can reject 99.5% of salt and show a flux rate of 51 LMH. In Reference [44], a similar effect was achieved for water with 35,000 mg/L in salt concentration but with a much lower pressure, at 55 bar [103].

Overall, TFC membranes are affordable, offer good performance, and are durable. However, their options for water treatment are limited, and they have some challenges with permeability, chlorine tolerance, fouling, and solvent resistance. From an alternative standpoint, researchers have explored the utilization of unconventional polymeric supports that possess remarkable properties. However, these materials tend to maintain a higher degree of hydrophobia. Consequently, investigations have focused on altering the membranes’ characteristics to enhance water affinity while preserving other advantageous properties. TFC membranes have more benefits compared to CA membranes. They can also reject some small organic molecules, remain stable in a broader range of pH levels, and work well at hotter temperatures.

Chlorine weakens these membranes, making them worse at rejection of salt. They can only handle a little chlorine exposure, unlike CA membranes. In this case, chlorine must be removed from the water before the use of TFC membranes [155]. Using specific monomers with -OH functional groups during polymerization, such as m-aminophenol and bisphenol-A, can enhance the resistance of the membrane to chlorine. At the same time, ester linkages decrease the number of sites available for chlorine attack [156]. The ability of CA membranes to reject salt decreases with higher temperatures; the feed water temperature should not exceed 35 °C [103]. Containing more salt makes it harder for the water to pass through the membrane. The water needs more pressure to permeate, leading to higher energy consumption [154]. Hermans et al. [157] examined the synthesis parameters and morphologies of PA layers formed on PSf supports via IP. Their investigation involved testing various additives in the aqueous phase, including meta-phenylenediamine (MPD), under filtration conditions with 1000 mg/L of MgSO_4_ and the pressure at 41 bar. The study revealed that combining sodium dodecyl sulfate (SDS) with a base-like TEA enhances membrane performance, leading to a superior water permeation rate of 1.4 LMH and MgSO_4_ rejection above 95%.

##### Thin-Film Nanocomposite (TFN)

**Figure 9:**
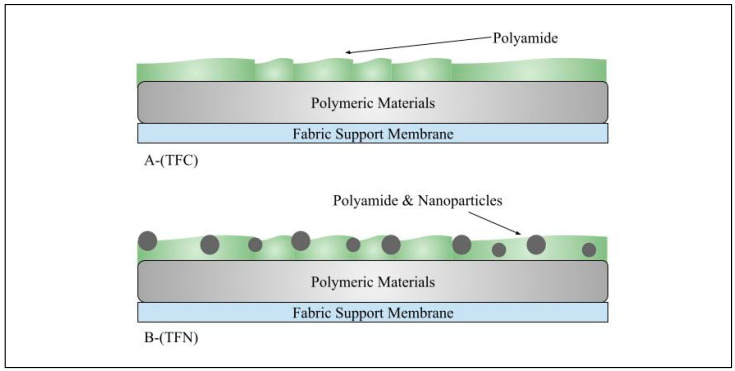
Concept of surface modifications: (**A**) TFC membrane; (**B**) TFN membrane (adapted from [165]).

Figure 9 illustrates the difference between TFC and TFN membranes. TFNs are an advancement of traditional TFCs created via IP. These modifications involve integrating nanoparticles into a thin polyamide (PA) layer at the surface of the TFC membrane to enhance its performance [158]. This enhancement may manifest in various ways, such as improved water permeability and solute rejection. The process of IP, which occurs between aqueous solutions of (PIP) or m-phenylenediamine (MPD) and organic solutions of TMC, is well-known for producing polyamide (PA). Jeong et al. [158] introduced a TFN membrane synthesized by incorporating zeolite NaA nanoparticles (0.004–0.4% *w*/*v*) into the PA layer. Integrating the zeolite nanoparticles while maintaining rejection properties similar to traditional (TFC) membranes introduced a significant improvement in membrane flux. Since then, TFNs have become helpful in creating NF and RO membranes for water treatment. Peeters et al. [159] also noted that membranes with a higher negative charge retain bivalent anions such as sulfate (SO_4_^=^) better than monovalent anions. A handful of research publications used other nanomaterials [160,161,162,163]. Hu et al. [164] discovered that increasing TMC concentration from 0.05 to 0.30 *w*/*v*% and extending the reaction time from 10 to 60 s during the interfacial polymerization significantly enhanced the separation performance of NF membranes for Na_2_SO_4_. The improvements were notable, with separation efficiency rising from about 93% to 97.5% and 96.5% to 97.5%, respectively. They also found that as the PIP concentration increases from 0.2 to 1.0% (*w*/*v*), Na_2_SO_4_ rejection improves while water flux decreases. Among the nanoparticles used for TFN/NF is silver (Ag), an anti-biofouling agent; it has become influential in permeate flux and MgSO_4_ rejection. The results indicated that the PA/Ag membranes achieved optimal performance with a flux of 92 LMH and a MgSO_4_ rejection rate of 97% at 14 bar with a 2000 mg/L sulfate concentration [129].

## 7. Sulfate Treatment Methods

Increasing concerns about elevated sulfate levels affecting water salinity are leading to the need for stricter regulations and sulfate treatment. Some sulfate treatment methods are being investigated and particles used for future development; the knowledge shared is described below in an overview. These methods have been categorized into the following groups: chemical treatment with precipitation of minerals, ion exchange, membrane contactors, EDR, evaporative techniques, and biological sulfate removal techniques, such as SRB [166]. Different treatment processes have been mentioned in this paper for review. The selected method is chosen based on its effectiveness in removing sulfates, the data availability, and its cost.

### 7.1. Chemical Precipitation

Gypsum precipitation has been a well-established method for treating highly acidic mine waters due to its effectiveness, simplicity, and tolerance towards temperature fluctuations [167]. While gypsum precipitation is effective and relatively simple, it might not be adequate in complying with environmental regulations when the water contains highly soluble metal sulfates such as sodium sulfate [168]. This process has some drawbacks, including the generation of large amounts of high-water-content sludge, challenges in sludge dewatering, high disposal costs, and equipment scaling [169]. In the process of gypsum precipitation for sulfate removal, an alkaline chemical such as lime, as shown in chemical reaction (1), is commonly used to raise the pH of mine water to around 9.5. Alternatively, limestone, as shown in chemical reaction (2), can be utilized, but it can only increase the pH to approximately 7 [170].

Ca(OH)_2_ (s) + H_2_SO_4_ (aq) → CaSO_4_·2H_2_O (s) (1)

Ca(CO_3_) (s) + H_2_SO_4_ (aq) + H_2_O → CaSO_4_·2H_2_O (s) + CO_2_ (g) (2)

The challenges in gypsum precipitation for sulfate removal can be addressed by using polymers like polyacrylamides and polyamines to improve dewatering and strengthen floc structure. Recycling a portion of the sludge through the high-density-sludge (HDS) process enhances settling and dewatering [171]. The solubility of gypsum imposes a minimum achievable sulfate concentration of around 1500 ppm at 20 °C in the absence of sodium or magnesium ions, but lime treatment at high pH overcomes the inhibitory effect of magnesium, enhancing gypsum precipitation [168]. Overall, these strategies improve the efficiency and effectiveness of sulfate removal through gypsum precipitation.

Ettringite precipitation has demonstrated the ability to reduce sulfate concentration to as low as 200 ppm [172,173]. But having magnesium in the treated water makes it harder to remove sulfate using the ettringite-precipitation method [174]. When dealing with water that has a high level of sulfates, over 3000 ppm, it can be cost-effective to use lime treatment first before using ettringite precipitation. This method helps decrease the requirement for costly aluminium-based chemicals. After ettringite precipitation, the pH of the treated water can be lowered by treating it with CO_2_ to meet discharge limits, resulting in the precipitation of calcite [175]. To minimize operational expenses in ettringite precipitation, the aluminium salt can be regenerated by decomposing the ettringite precipitated or by exploring alternative applications for the precipitate, such as using it as a substance to capture and remove arsenate [176,177]. An associated chemical reaction (3) is shown below.

6Ca(OH)_2_ + 3H_2_SO_4_ + 2Al(OH)_3_ (s) + 20H_2_O → Ca_6_Al_2_(SO_4_)3(OH)_12_·26H_2_O (s) (3)

In hydrometallurgical processes, it is common to use jarosite precipitation for the purpose of removing iron, as represented by the chemical compound with a combination of Na^+^ or K^+^ shown in chemical reaction (4) [178]. Jarosite, a natural secondary mineral found in acid mine drainage, requires low pH, a high temperature, high pressure, and a specific reaction time [179]. However, the dissolution of jarosite under decreased acidity hinders its effectiveness. In consequence, jarosite is not practical for sulfate removal due to poor results and challenging reaction conditions [180].

3Fe_2_(SO_4_)3 (aq) + Na_2_SO_4_ (aq) + 12H_2_O → 2NaFe_3_(SO_4_)2(OH)6 (s) + 6H_2_SO_4_ (aq) (4)

Sulfate precipitation through barite can be achieved by using barium salts such as barium- hydroxide, sulfide, or carbonate [181]. Barium- hydroxide, and sulfide can be directly used to treat extremely acidic mine waters, whereas barium carbonate needs to be treated with lime first [182]. When using barium sulfide, it is essential to remove hydrogen sulfide through a stripping process [183]. The use of barite precipitation in sulfate removal from mine water has resulted in sulfate concentrations lower than 200 ppm [182,183]. Barite has very low solubility, but the precipitants themselves are costly and toxic. Furthermore, this procedure produces waste containing barium, which requires appropriate disposal or, if possible, recycling [184]. One approach for recycling involves heating BaSO_4_ to make BaS and then turning it into BaCO_3_ and H_2_S by adding CO_2_ to a solution of BaS [185]. The relevant chemical reactions ((5)–(7)) are shown below.

Ba(OH)_2_ (s) + H_2_SO_4_ (aq) ⇌ BaSO_4_ (s) + 2H_2_O (5)

BaS (s) + H_2_SO_4_ (aq) ⇌ BaSO_4_ (s) + H_2_S (g) (6)

BaCO_3_ (s) + H_2_SO_4_ (aq) ⇌ BaSO_4_ (s) + H_2_CO_3_ (aq) (7)

The use of limestone in water treatment is considered an improvement over conventional lime treatment. Limestone beds, commonly known as anoxic limestone drains (ALDs) are constructed without any contact with the atmosphere [186]. The main purpose of adding limestone is to introduce alkalinity to the solution, which helps to neutralize the AMD. This approach has been studied and implemented by researchers [187,188,189]. The lime treatment method requires additional management of the sludge produced from the precipitation of calcium salts. In contrast, the use of limestone promotes the sorption of sulfate on the surface of the limestone, resulting in a minimal need for maintenance [190,191]. The solubility of limestone is influenced by factors such as temperature, pH, and CO_2_ concentration [188]. Using a limestone fluidized bed for treatment can result in cost savings of 29–38% compared to lime, even though the capital costs remain unchanged [192]. A study suggested combining the limestone process with lime treatment yields improved results. In their research, the goal was to decrease sulfate levels from 3000 to 1200 ppm. They successfully achieved this by first neutralizing the water with limestone, which reduced sulfate levels to 1900 ppm [168]. Likewise, another study reported a significant decrease in sulfate levels from 15,000 to 2000 ppm through the use of limestone neutralization [193]. However, it is important to carefully monitor the gypsum saturation level because when it becomes saturated, limestone dissolution stops. Appropriate pre-treatment is essential; the wastewater from limestone treatment can be combined with another treatment method to improve the overall outcomes. On the other hand, if the sulfate loading exceeds the limits for gypsum precipitation considerably, lime treatment is still preferred due to its cost-effectiveness and high efficiency [193]. Overall, the use of lime for treating AMD can be an economical method and has proven effective in precipitating sulfates from contaminated water. However, the application of limestone, as an alternative to conventional lime treatment, is considered an advancement in the treatment process [186].

### 7.2. Ion Exchange

**Figure 10:**
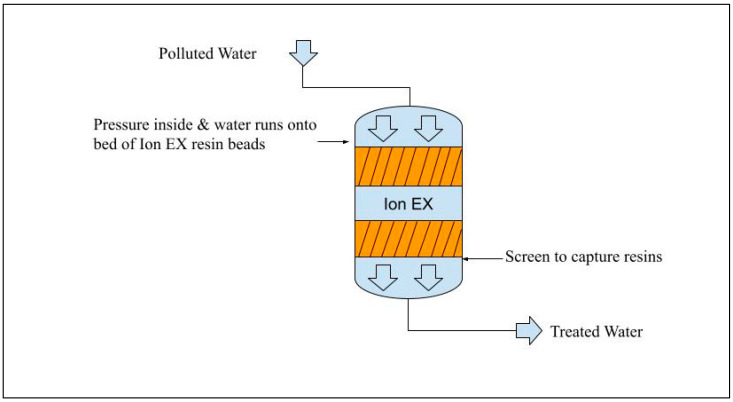
Process of ion-exchange treatment (adapted from [195]).

Ion-exchange resins can remove ions from solutions by exchanging similar ions on the resin surface. Cation exchange resins are commonly used for water softening by exchanging calcium and magnesium ions in water with sodium ions on the resin surface [194]. Ion exchange is the most widely used method for removing large quantities of sulfate from water for commercial and public supply [194]. Figure 10 represents the ion-exchange process. This process involves rinsing the resin, once it is loaded to capacity with sulfate, with a concentrated salt solution to regenerate it and then remove the sulfate using a concentrated brine solution [194,195]. There are several sulfate-specific ion-exchange technologies available on the market, such as Sulfate IXTM and GYP-CIX [196]. Furthermore, ion exchange for sulfate can also remove other anions such as nitrate.

Ion exchange has some drawbacks, including the requirement to properly dispose of the concentrated regeneration solution and the need to eliminate solids and organic matter upstream to prevent fouling of the resin [194]. When using ion exchange to clean water with 500 ppm sulfate, the goal is to have treated water with sulfate levels ideally below 200 ppm, provided that the process is carried out correctly. During the regeneration process, concentrated liquid salt waste (brine), which contains a high concentration of sulfate, is produced. This brine waste, which makes up about 2–5% of the initial flow, must be disposed of properly [197]. In the Sulf-IX system, a specialized method is used to chemically precipitate and remove sulfate from the concentrated brine solution [197].

### 7.3. Membrane Contactor (MC)

**Figure 11:**
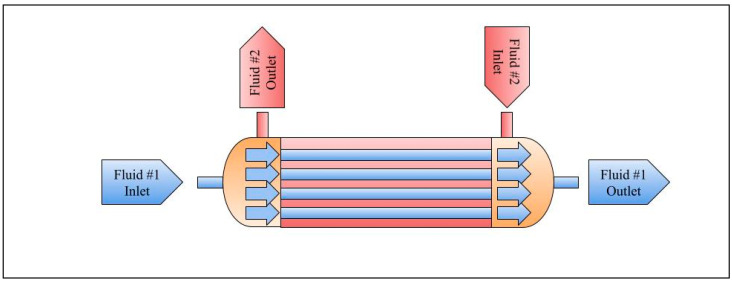
Hollow fiber membrane contactor (adapted from [198]).

A membrane contactor (MC) is an advanced system that combines multiple processes like absorption, desorption, and extraction in a single unit, offering improved efficiency compared to traditional methods like tray towers and packed beds. Conventional contactors require phase separation, but membrane contactors allow for non-dispersive phases to interact, reducing common issues like flooding, channeling, and foaming [102,198]. Furthermore, membrane contactors provide a larger cross-sectional surface area between the two surfaces, which improves mass transfer efficiency and results in more efficient separation systems as the main function. The volume of equipment required for liquid extraction can be reduced by over 500 times. The MC is generally designed as a hollow-fiber module, as illustrated in Figure 11. The mechanism was described in Reference [198]. MC is used for separations, such as gas/liquid or liquid/liquid, involving either porous or nonporous membranes. These processes are useful for applications such as membrane crystallization, membrane gas transfer, pervaporation, membrane distillation, facilitated transport membranes, membrane emulsification, and osmotic distillation [102].

Recently, advanced applications introduced the term zero liquid discharge (ZLD). Drioli et al. [199] studied five integrated systems combining membrane crystallization (MCr) with RO, NF, or MF. They found that using NF or RO beforehand reduces water hardness and removes multivalent ions. Sulfate salts in the concentrated brine are treated as a resource for crystal production instead of waste. In addition, water recovery was achieved at 92.8% without increased operational costs. In another study, Drioli et al. [200] investigated the integration of a membrane unit successfully recovering solid products like CaCO_3_, NaCl, and MgSO_4_·7H_2_O from the nanofiltration rejection. In both studies, sodium carbonate was added to remove calcium ions by forming calcium carbonate, which prevents scaling from calcium sulfate and ensures efficient magnesium sulfate recovery. This method is an effective and economical solution for sulfate removal in water treatment. Likewise, Gomez et al. [201] explored the use of (MCr) to extract copper sulfate from acidic mine waters, providing a sustainable approach to manage industrial waste. Frappa et al. [202] examined membrane condensers, as a recent innovation in membrane contactor technology, as to their ability to remove contaminants from waste gaseous streams like NH_3_, HF, and SO_2_. The results show a significant rejection rate, and water recovery is achieved based on temperature and humidity. Dow et al. [203] studied a direct contact membrane distillation (DCMD) pilot system for treating textile wastewater in Australia. The system operated for 90 days without membrane wetting, with initial water flux at 5 LMH, which then decreased to 2 LMH after 65 days. Using cleaning restored 79% of the original flux. In addition, the rejection of the non-volatile sulfate was over 99.9%, and water recovery reached 91.6%. This system achieved zero liquid discharge.

### 7.4. Evaporative

**Figure 12:**
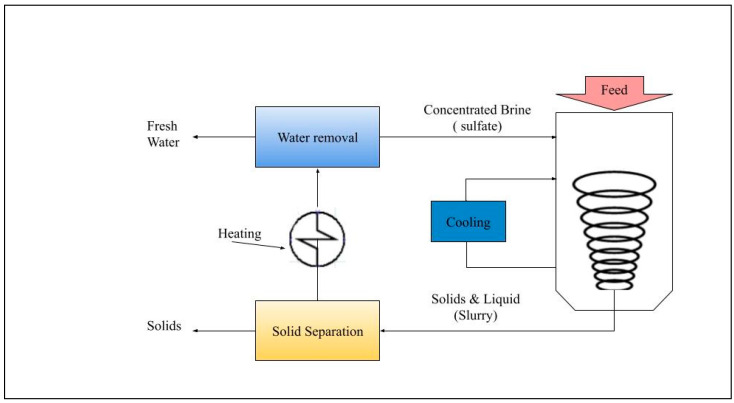
Process of ZLD system (adapted from [204]).

Evaporative treatment is another alternative for removing sulfate from water. This method involves evaporating the water to leave behind concentrated brine or solid residuals, such as salts, which cannot be evaporated. The evaporated water can be reused. Nevertheless, evaporative methods, also called zero-liquid discharge (ZLD) technologies, are usually used in the power and refining sectors rather than for municipal wastewater treatment. This is primarily due to the considerable initial costs, ongoing operation expenses, and maintenance burdens linked to these techniques [166]. In a standard ZLD process, of which some stages are provided in Figure 12, the outcome is usually a solid salt, with no liquid, that requires proper disposal at a suitable facility. In the ZLD process, the ways to handle the concentrated membrane solution include using thermal or mechanical evaporation.

In the ZLD technology, there is a solid contact clarifier, and ultrafiltration is involved. Before this, the pre-treatment involves using lime–soda ash softening, and a polymer is introduced to improve the settling of solids. To prevent scale formation on the ultrafiltration (UF) membranes, sulphuric acid and polyphosphate are added. UF disposes of the remaining particles in the water. After this, the UF filtrate undergoes disinfection before being combined with the current of the water treatment plant permeate, making it safe for drinking-water distribution [205]. Municipalities seldom require treatment along the lines of the parameters that would necessitate this technology. Furthermore, evaporative treatment is energy-intensive, as it requires considerable energy to evaporate water. This high energy demand translates to a high cost of approximately $10 to $20 per 1000 gallons of treated water [206]. The concentrated brine or salt solids produced from the process also require proper disposal. As a result, evaporative technologies are usually mechanically complex and require materials resistant to corrosion, making them expensive to set up.

### 7.5. Sulfate Reducing Bacteria (SRB)

Bacteria have the ability to respire using sulfate in the absence of oxygen. This biological sulfate removal process involves reducing sulfate to sulfide using bacteria. The sulfide can then be eliminated by precipitating it with metals in the water or releasing it as hydrogen sulfide gas into the atmosphere. An organic material or bacterial substrate is required as a food source in this process. The addition of metals may also be necessary to remove sulfide from the water phase [207,208]. Constructed wetlands or bioreactors can be utilized for biological sulfate removal, and it has proven to be effective in the mining industry for removing sulfate from mining-impacted waters. Constructed wetlands can lower sulfate levels to 250 ppm [209].

SRBs are highly beneficial for bioremediation due to their high practicality, resource recovery, low cost, and low pollution [210]. On the other hand, the biological sulfate reduction process has certain drawbacks associated with it. These include slow process kinetics, the need for organic compounds as electron donors, an increase in dissolved organic content in the treated effluent [211,212], and inhibition by high salinity and metal ions [174]. This process also results in the creation of hydrogen sulfide, a harmful gas [174]. Additionally, certain organic materials utilized as electron donors can add color to the wastewater [213]. From a phylogenetic perspective, SRB can be categorized into three primary branches, which consist of the cold, mesophilic, and thermophilic species [214]. These branches are comprised of the δ-subclass of proteobacteria, Gram-positive bacteria such as Desulfosporosinus and Desulfotomaculum, and a branch formed by Thermodesulfovibrio and Thermodesulfobacterium. SRB can utilize a wide range of carbon and energy sources, encompassing hydrogen, formate, lactic acid, glycerol, and ethanol. Bioreactors maintain SRB growth by using cheap carbon sources such as wood chips, hay, compost, sludge, and sawdust [215]. The optimal pH range for the activity of SRB is between 5 and 9. At pH conditions outside of this range, SRB’s activity rate will be reduced [216].

According to [217] the metabolic process of SRB is quite complex due to the involvement of numerous biochemical reactions catalyzed by various enzymes. The activity of SRB in bioremediation is significantly affected by temperature conditions, with an optimal range of 1–8 °C and reduced activity at high temperatures [214,218]. SRB can assist in making polluted streams less harmful and slowing down the spread of contaminants by reducing metal redox states and facilitating the precipitation of metal sulfides [214]. Utilizing gaseous substances as sources of electrons in sulfate reduction treatment is advantageous in avoiding wastewater dilution and secondary pollution from unused donors [219]. In contrast, Minnesota wastewater treatment facilities in the US declined to adopt biological sulfate removal due to its unreliable performance and the requirement of the addition of carbon. Numerous experimental studies have verified that the use of SRB in bioremediation is an efficient approach for treating AMD [219,220]. Ferrous sulfate bacteria can oxidize ferrous Fe^2+^ in AMD to ferric Fe^3+^ under acidic and oxygen-rich conditions, leading to the removal of heavy metal ions such as ferrous Fe^2+^ from water. This process can also result in the precipitation of polymeric ferric sulfate and ferric oxide, which can aid in the removal of heavy metal ions and sulfate from the water [216].

### 7.6. Electrodialysis (ED and EDR)

**Figure 13:**
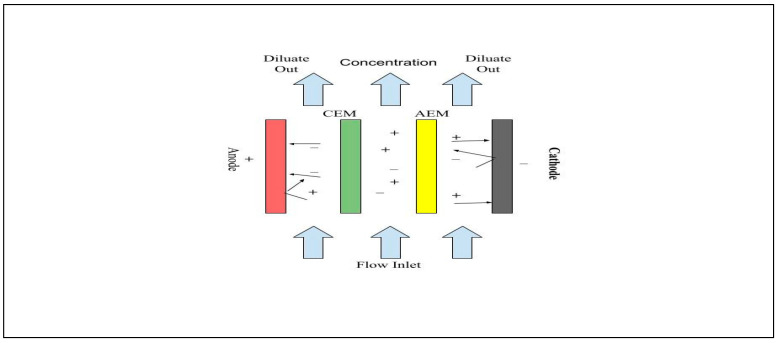
Diagram of the process of ED (adapted from [223]).

Electrodialysis (ED) is a method of separation and purification that utilizes electrical current as a driving force. The process involves placing alternating anionic and cationic membranes between the cathode and anode in the electro-dialysis module. The process of ED is described in Figure 13**.** When activated, the positively charged cations move toward the cathode, while the negatively charged anions move toward the anode, leading to the creation of fresh water and concentrated water, respectively [216]. One of the advantages of ED is that it does not require any additional chemicals and can operate continuously [221]. ED technology has broad applications in various industries, including wastewater, acid production, electroplating, and seawater. A separate study evaluated the potential of ED in recovering water from AMD [222].

Electrodialysis reversal (EDR) is a technique that functions in a manner similar to electrodialysis, but with periodic reversal of electrode polarity and automatic exchange of concentrated and diluted solutions. In EDR, electricity is used to move dissolved salt ions through charged membranes [224,225]. However, it should be noted that EDR is not effective in eliminating pathogens, suspended solids, or uncharged compounds [226]. Originally developed in the 1970s, EDR has found significant application in desalination projects across the Middle East and other regions [227]. While EDR has not been employed for wastewater treatment in Minnesota, it has been successfully utilized for treating wastewater in California as part of water reuse initiatives [228]. The EDR method employs electrical energy to drive water through the membrane. Before being fed into an EDR system, water should undergo a pre-treatment such as MF or sand filtration to eliminate suspended solids and organic substances that can potentially reduce the lifespan of the EDR equipment [194]. Compared to RO membrane filtration, EDR can treat water with higher salt and organic material concentrations, although its removal efficiency is lower [227].

EDR operation is more intricate than membrane filtration [194]. However, it provides benefits such as greater water recovery and decreased management demands for brine solutions. ED and EDR offer a distinct advantage in treating agricultural water by selectively removing the most detrimental salts, such as chloride ions. In an EDR pilot plant that treated municipal wastewater for agricultural purposes, the TDS in the influent was reduced by 71%, decreasing from 1104 ppm to 328 ppm [229]. Moreover, pilot systems utilizing ED for wastewater treatment have consistently achieved the required water quality standards for durations of over six months [230].

Most of the treatment methods have the ability to remove sulfate efficiently, with different percentages of efficacy. Chemical precipitation is economically viable for mining but results in notable waste production and reduces sulfate concentrations within a range of 100 to 1200 ppm. Membrane contactor technology offers novel possibilities for designing and optimizing sulfate treatment but faces challenges in mass transfer resistance. In comparison, biological treatments have lower energy and material needs, making them less expensive, but require long rejection times and depend on factors like the time of year and environmental conditions. In contrast, evaporative and electrodialysis technologies have expensive setup, operation, and maintenance costs. Ion-exchange methods are unsuitable for treating mine water unless significant pre-treatment is performed.

## 8. Flux and Rejection

To enhance permeate flux and rejection of sulfate, high turbulence is generated with high CFV in cross-flow filtration. As mentioned in Section 5, this also involves minimizing concentration polarization at the membrane surface. This significantly inhibits the formation of a cake layer. Dead-end filtration can experience drops in the permeate flux. Pressure levels, hydrophilic degree, and pore size possess high effectiveness in manipulating rejection levels and the altering the flux of membranes. Experiments were carried out at various pressure values for pristine membranes, TFC membranes, and TFN membranes. The flux and rejection were shown to be in a trade-off relation. Researchers observed that when increasing the pressure, the flux would increase regardless of nanoparticle loads since the pressure acts as a driving force causing much rejection or brine within a short time inside the membrane [231]. Nevertheless, the membrane’s hydrophilicity can enhance the flux with increased nanoparticle loads due to functionalization groups such as COOH and OH. Flux and rejection were measured at the specific values of nanoparticles loaded, and these mechanisms were observed relative to the pore size of the membrane (morphology) and the formation of small cells in structures with large pores and macro voids [97]. Large pores provide high permeate flux by substituting DI water and solvent through phase inversion. The membrane matrix interacted less with nanoparticles, increasing the flux according to its hydrophilicity. Increasing nanoparticles may increase contact angles created by the van der Waals force between the nanoparticles and membrane, and when nanoparticle loads increase, this can generate high density and form hydrophobic areas and smaller pore sizes due to an increase in viscosity and decrease in polymer volume; this summarizes the consequences of the reduction in flux as discussed by Crescenzo et al. [232]. In other words, with increasing nanoparticle loads, the flux decreases, and an accumulation is formed [233]. Experimentation at lower pressure values indicated high rejection due to the increase in contact time for the solute and the membrane. In contrast, the rejection decreases, with an incremental driving force, the pressure produced [97]. The rejection can be increased most through increases in the size of the molecule. As explained by the Donnan exclusion phenomenon, the membrane surface charge significantly increases or decreases the flux and rejection [234]. The flux and rejection are linked to each other in order to have a balanced system. As the solution pH increases, negatively charged membranes tend to have a higher surface charge, whereas positively charged membranes exhibit the opposite trend [235,236].

Moreover, the presence of divalent cations in a solution tends to decrease the negative surface charge of a membrane [236,237]. Negatively charged membranes contain negative fixed charges on the polymer backbone, typically attributed to functional groups such as carboxylic or sulfonic acid. Specific NF membranes exhibit amphoteric characteristics, meaning their charge can be either positive or negative depending on the pH of the surrounding environment [238]. In addition, some NF membranes in the market have both acidic and essential parts so that they can behave differently depending on the pH of the solution. At a certain pH, called the isoelectric point, the membranes become neutral. This flexibility allows them to work well in various pH conditions [159,235,236,239]. This charge property of NF membranes is an essential factor influencing their separation performance and must be considered in membrane selection and application [113,235,236,239]. The charge of membranes affects how well they retain ionic species. The ion-exchange capacity tells us about the overall charge of the membrane, especially for RO, while the surface charge is more critical for nanofiltration NF membranes [235]. The surface charge influences ions’ retention and fouling substances’ attraction to the membrane [112,114]. We can measure the surface charge by examining the streaming potential, which helps understand fouling and determine the zeta potential at the membrane surface [235]. Negatively charged membranes have weakly acidic functional groups, such as carboxylic acids [235,236]. The membrane’s surface charge can be modified by adsorbing charged surfactants or organic macromolecules like humic acids.

For example, the rejection of simple ionic components in the NF membrane is primarily determined by the membrane’s inherent charge. Non-charged membranes do not exert electrostatic forces on simple ionic components. Hence, in their rejection of simple salts, for instance, the order is typically governed by molecular size or diffusion coefficient; Na_2_SO_4_ is bulkier than chloride CaCl_2_, which is more significant than NaCl. Because of electrostatic interactions, positively charged membranes repel positively charged ions, particularly divalent cations, while attracting negatively charged ions, especially divalent anions. Consequently, the rejection order is reversed for a positively charged membrane (CaCl_2_ higher than NaCl higher than Na_2_SO_4_). The negatively charged membranes tend to repel negatively charged ions (e.g., SO_4_^=^) while attracting positively charged ions (e.g., Ca^2+^), resulting in this specific rejection order. For negatively charged membranes, the rejection order is generally as follows: Na_2_SO_4_ is higher than NaCl, which is in turn higher than CaCl_2_. Repelled divalent anions (e.g., SO_4_^2^) have lower rejection, and monovalent anions (e.g., Cl^−^) are moderately rejected. As for divalent cations (e.g., Ca^2+^), they are attracted and have higher rejection than monovalent cations (e.g., Na^+^), which are moderately rejected [159,240].

## 9. Membrane Fouling and Cleaning

References [86,203] used a chemical solution for cleaning sulfate salts. This method may have a negative impact on drinking-water-based applications. Fouling is determinative as to the life of the membrane and deteriorates the quality of the permeate. Three primary factors influence membrane fouling: operating conditions, feed characteristics, and membrane properties. Operating conditions significantly impact the fouling rate. It is crucial to optimize hydrodynamic conditions such as transmembrane pressure (TMP), cross-flow velocity (CFV), temperature, and pH to balance flux decline and fouling while optimizing rejection rates. The accumulation of solid particles, both suspended and dissolved, on the membrane surface happens because of what is called concentration polarization. Water flow along the membrane creates a layer in which water moving toward the membrane is moving faster than the particles diffusing away from it [152]. The resulting buildup, known as fouling or scaling, leads to higher operational costs. Cleaning chemicals and anti-scalants are needed, the membrane’s lifespan decreases, and water purification efficiency decreases [241]. Membrane fouling can be divided into inorganic, organic, and biofouling. Inorganic fouling occurs when scales like BaSO_4_ and CaSO_4_ are dissolved in the feed water; it restricts water flow and reduces the permeate flux. Most studies (72%) utilized synthetic water, 23% used real water, and 5% did not state the water source [25]. Organic fouling happens when natural organic materials such as humic acids, proteins, and carbohydrates deposit on the membrane, forming a layer that decreases permeability and flux. Biofouling is caused by microorganisms attaching to the membrane and forming biofilms, which resist water flow and degrade performance, particularly in environments with high microbial activity, such as NF systems [242,243,244].

Choosing the suitable membrane and operating conditions can minimize fouling, but sometimes cleaning is needed to maintain peak performance. Cleaning should happen if the permeate flux drops by more than 10%, the salt content in the permeate rises by 10%, or the normalized pressure drops by 15% compared to ideal conditions. Cleaning frequency can vary depending on the application, from days to months [245]. Membrane cleaning, utilizing either physical or chemical treatments, is essential to fouling-control strategies. Typically, chemical treatment and cleaning involve two steps: rinsing with clean water to replace feed water and applying the cleaning agent. Afterward, another round of rinsing ensures a thorough cleaning. Chemical-free cleaning in membranes and turbulence washing are typically limited to clean water. This process involves pumping clean water at a high flow rate to achieve a slight flux recovery. Different types of foulants require specific cleaning agents: acids for mineral deposits and salts; alkalis for organic foulants; enzymes for microorganisms; and detergents for oils, fats, and grease. Tubular systems utilize sponge balls to effectively clean all types of foulant [49]. Zoubiek and Henni [246] tested three cleaning chemical solutions, including acidic, alkaline, and surfactant solutions, in various combinations to explore the best means of effective cleaning using a solution. In general, an acidic chemical cleaning solution formed by a combination of (KOH/HNO_3_/H_3_PO_4_) leads to the highest flux recovery, at above 98%.

Physical methods such as flow reversal, where water flows back to the feed side from the permeate side, can also be employed. However, this method may compromise the membrane’s integrity and stability. The most common physical antifouling treatment methods are primarily based on hydrodynamic principles. They include forward and reverse flushing, backflushing, back pulsing, and surface shearing. We recently introduced a physical treatment for fouling control named the periodic transmembrane pressure technique (PTMP). Zoubeik et al. [247] also explored a novel antifouling technique called the periodic feed pressure technique (PFPT). Experimental results indicate that PFPT effectively reduces fouling, improves filtration performance, and maintains membrane permeability, offering a promising solution for enhancing the efficiency and durability of polymeric membranes. Similarly, Echakouri et al. [248] studied three physical treatments to mitigate fouling: periodic transmembrane pressure (PTP), pulse, and backflushing. It found that the PTP technique was the most effective in maintaining membrane permeability and reducing fouling, followed by pulse, flow, and backflushing. This suggests that PTP could significantly enhance the efficiency and lifespan of membranes.

## 10. Conclusions and Future Recommendations

Sulfate is a concerning contaminant in both surface and groundwaters used for drinking. The World Health Organization (WHO) recommends a maximum sulfate concentration of 500 mg/L in drinking water. When present at high concentrations, it can cause dehydration, gastrointestinal irritation, and laxative effects in animals or humans. This concern encompasses various fields, such as agriculture and aquatic life, leading to low productivity and cattle deaths. Corrosion, scale, and plugging due to sulfated water can also occur in the oil and gas industry. Due to its effectiveness and versatility, membrane technology is considered one of the most promising methods for the purification of water. Membranes are particularly efficient at removing sulfates and other contaminants. Researchers have developed numerous methods to enhance the filtration process. Membranes based on polymeric materials have continuously evolved, with ongoing research being conducted into various modification techniques and compositions. For instance, different nanomaterials have been added to the surfaces of polymeric membranes to improve their performance and antifouling properties. Modified polymeric membranes incorporating hybrid nanoparticles have demonstrated excellent sulfate rejection, as shown in the innovative membranes developed by Wan Azelee et al. [249]. Hydrophobic membranes commonly remove organic contaminants such as fat, oils, and synthetic solutions. Hydrophilic membranes offer high permeate flux and reduced fouling and are typically used to remove sulfate salts, proteins, and sugars. Commercially available hydrophilic membranes include CA and PA. Our review focused on exploring new materials, and our findings indicate that PSf and PES membranes, when integrated with the nanoparticles, are the preferred choices for removing a wide range of sulfate salts. However, it was observed that these materials did not perform well with silica nanoparticles for sulfate removal. Silica-based studies were found to have high rejection rates for Na_2_SO_4_ but lower rates for MgSO_4_.

On the other hand, PVDF membranes have shown high levels of sulfate rejection by incorporating nanoparticles (e.g., ZnO or TiO_2_). Nevertheless, PVDF membranes are typically recognized for having a negatively charged surface, attributed to the presence of C-F groups [250]. This fact does not prevent the use of other nanoparticles, both inorganic (e.g., Ag, Fe_3_O_4_) and organic (e.g., GO, CNTs), which possess high surface area, low mass, and low mechanical, and thermal resistances. Despite their benefits, hydrophilic nanoparticles can leach into both permeate and rejection, causing environmental concerns that require more research. Most polymer-based membranes experience flux decline and maintain lower steady-state permeate flux than modified membranes. Fouling can occur within the membrane, on its surface, or both. Internal fouling involves the deposition and adsorption of particles inside the membrane pores, while external fouling forms a cake layer on the membrane surface. Physical cleaning is preferable to chemical treatment. Chemicals employed to minimize fouling or scaling can end up in freshwater sources, leading to water pollution. No perfect membrane exists; each type has unique features and optimizes different parameters. The industry continues searching for a membrane with high efficiency (high flux and rejection rate) and low cost. The ultimate goal is to develop a perfect membrane that experiences no fouling and maintains high flux. However, future research must also explore the potential risk of nanoparticles and determine the extent to which they may leach into the waters treated for sulfates.

## Figures and Tables

**Table 1:** Properties of various types of membrane module designs [104].

Property	Spiral/Wound	Flat/Plate	Tubular	Hollow/Fiber
Packing density (m^2^/m^3^)	500–1000	200–500	70–100	500–5000
Manufacturing cost	Moderate	High	High	Low
Ease of cleaning	Poor to good	Good	Excellent	Poor
Energy demand	Moderate	Low to moderate	High	Low
Fouling potential	High	Moderate	Low	Very high

**Table 2:** Characteristics of different membranes [103,111].

Membrane Process	MF	UF	NF	RO
Pore size	50–10,000 nm	5–100 nm	1–10 nm	<2 nm
Membrane structure	Asymmetric or symmetric, porous	Asymmetric, microporous	Asymmetric, thin-film composite, tight porous	Asymmetric, thin-film composite, semi-porous
MWCO	>200,000 Da	1000–200,000 Da	200–1000 Da	>100 Da
Retained	Bacteria, colloids, organics, suspended solids	Proteins, oils, lactose, vitamins, organic	Divalent: anions and cations, organics	Monovalent ions, all contaminants
Thickness surface film	10–150 µm	150–250 µm	150 µm	150 µm
Average permeability	500 (L/m^2^ h bar)	150 (L/m^2^ h bar)	10–20 (L/m^2^ h bar)	5–10 (L/m^2^ h bar)
Filtration mechanism	Molecular sieve	Molecular sieve	Solution diffusion	Solution diffusion
Membrane materials	PES, PSf, PA, PP	PVDF, PES, PP, PAN	CA, PA, PI, SPSU	CA, PA, PI, SPSU
Pressure	0.1–3 bar	2–4 bar	5–40 bar	7–100 bar

## Data Availability

All data are presented in the article.

## Abbreviations

AL_2_O_3_	Nano-sized alumina
Ag	Silver
AMD	Acid mine drainage
Au	Gold
BaSO_4_	Barium sulfate
BDSA	Benzidinedisulfonic acid
BHTTM	Bis(1-hydroxyl-1-trifluoromethyl-2,2,2-trifluoroethyl)-4,4′-methylenedianiline
CA	Cellulose acetate
CaSO4	Calcium sulfate
CdSO4	Cadmium sulfate
CFFO	Carboxyl functionalized ferroferric oxide
CFV	Cross-flow velocity
CMC	Carboxymethyl chitosan
CNTs	Carbon nanotubes
CuSO_4_	Copper sulfate
DMAc	Dimethylacetamide
DMF	Dimethylformamide
ED	Electrodialysis
EDR	Electrodialysis reversal
Fe_2_O_3_	Ferric oxide
GE	General Electric
GO	Graphene oxide
HACC	Hydroxypropyl trimethyl ammonium chloride chitosan
HNT	Halloysite nanotubes
HPEI	Hyperbranched polyethyleneimine
IP	Interfacial polymerization
K2SO_4_	Potassium sulfate
KOH/HNO_3_/H_3_PO_4_	Potassium hydroxide+ Nitric+ Phosphoric acids
KDa	Kilodalton
LIPS	Liquid-induced phase inversion
MC	Membrane contactor
LMH	Permeate flux
MF	Microfiltration
MgSO_4_	Magnesium sulfate
MMMs	Mixed matrix membranes
MOF	Metal–organic frameworks
MPD	Meta-phenylenediamine
MWCO	Molecular weight cut-off
NaSO4	Sodium sulfate
NF	Nanofiltration
NIPS	Non-solvent-induced phase inversion
NMP	N-methyl pyrrolidone
PA	Polyamide
PEG	Polyethylene glycol
PES	Polyethersulfone
PFPT	Periodic feed pressure technique
PI	Polyimide
PIP	Piperazine
PP	Polypropylene
PPEA	Poly (ethylene glycol) phenyl ether acrylate
PS	Phase separation
PSf	Polysulfone
PTMP	Periodic transmembrane pressure technique
PTP	Periodic transmembrane pressure
PVC	Polyvinyl chloride
PVDF	Polyvinylidene fluoride
PVP	Polyvinylpyrrolidone
PWF	Pure-water flux
RO	Reverse osmosis
SDS	Sodium dodecyl sulfate
SPES	Sulfonated polyether sulfone
SPSU	Sulfonated polysulfone
SRB	Sulfate reducing bacteria
SWCNTs	Single-walled carbon nanotubes
TEA	Triethylamine
TFC	Thin-film composite
TFN	Thin-film nanocomposite
TiO_2_	Titanium dioxide
TIPS	Thermal-induced phase inversion
TMC	Trimesoyl chloride
TMP	Transmembrane pressure
UF	Ultrafiltration
VIPS	Vapor-induced phase inversion
WHO	World Health Organization
ZnO	Zinc oxide
ZLD	Zero liquid discharge
